# Interfacing vector-borne disease dynamics with climate change: Implications for the attainment of SDGs in Masvingo city, Zimbabwe

**DOI:** 10.4102/jamba.v13i1.1175

**Published:** 2021-09-28

**Authors:** Lazarus Chapungu, Godwell Nhamo

**Affiliations:** 1 College of Economics and Management Sciences, Institute of Corporate Citizenship, Exxaro Chair of Climate and Sustainability Transitions, University of South Africa, Pretoria, South Africa

**Keywords:** climate change, adaptation, vector-borne diseases (VBDs), malaria, SDGs, communities, Masvingo, Zimbabwe

## Abstract

This study used a mixed-methods research design to examine the sensitivity of vector-borne disease (VBD) patterns to the changes in rainfall and temperature trends. The research focused on malaria in Masvingo Province, Zimbabwe. The study interfaced the climate action, health and sustainable cities and communities with sustainable development goals (SDGs). Historical climate and epidemiological data were used to compute the correlations and determine the possible modifications of disease patterns. Clustered random and chain-referral sampling approaches were used to select study sites and respondents. Primary data were gathered through a questionnaire survey (*n* = 191), interviews and focus group discussions, with Mann–Kendal trend tests performed using XLSTAT 2020. The results show a positive correlation between malaria prevalence rates and temperature-related variables. A decline in precipitation-related variables, specifically mean monthly precipitation (MMP), was associated with an increase in malaria prevalence. These observations were confirmed by the views of the respondents, which show that climate change has a bearing on malaria spatial and temporal dynamics in Masvingo Province. The study concludes that climate change plays a contributory role in VBD dynamics, thereby impeding the attainment of the 2030 Agenda for Sustainable Development, especially SDG 3, which deals with health. The study recommends further research into appropriate adaptation mechanisms to increase the resilience of rural and urban communities against the negative transmutations associated with weather and climatic pressures.

## Introduction

Climate change significantly influences the lives (Heshmati 2021), health and livelihoods (Ding et al. 2021) of people. A substantial body of scientific opinion (Ding et al. 2021; Fouque & Reeder 2019; Rocklöv & Dubrow 2020; Thomson et al. 2018; Wilcox et al. 2019) indicates that climate change is intricately involved in the manipulation of vector-borne disease dynamics. Over the years, scientists have made considerable progress in combating the proliferation of vector-borne diseases (VBDs), specifically malaria (Thomson et al. 2018). As a result, there has been a noticeable decline in the global mortality associated with VBDs. However, vulnerable communities in developing countries are still experiencing high incidences of VBDs. These can be ascribed to their susceptibility of such communities to VBDs as a result of poverty, ecosystem fragility, suboptimal health management systems and other factors (Rocklöv & Dubrow 2020). The spatial distribution of VBDs is a function of various socio-environmental factors that work together in a complex way. The ongoing long-term changes in weather patterns further modify the dynamics of VBDs. Thomson et al. (2018) observe that several human diseases are sensitive to climate, especially temperature and precipitation regimes. Consequently, a change in climatic variables will likely modify disease patterns as they influence the geography and biology of biting insects that transmit pathogens (Brand & Keeling 2017). Mordecai et al. (2020:417) state that ‘as climate change driven warming of the earth–atmospheric system occurs, VBD prevalence either increases or decreases relative to the thermal optima for disease transmission’. Broadly speaking, biological processes are accelerated by high temperatures. Thus it is expected that the warmer it gets, the faster the biological processes will change, ushering in several and diverse modifications, including modifications to infectious disease patterns (Byrd et al. 2020). Consequently, it becomes difficult to achieve sustainable development goals (SDGs) in regions already plagued by other socioeconomic problems. This interface brings to the fore perspectives on the 2030 Agenda for Sustainable Development (AfSD) and its aligned 17 SDGs, especially SDG 3 (health and wellbeing), SDG 11 (sustainable cities and communities) and SDG 13 (climate action). All these perspectives call for communities to adapt to the changing climate and disease patterns. The selected applicable targets from the SDGs in focus are shown in Box 1.

BOX 1 Selected provisions of the focus sustainable development goals.

SDG 3 – Ensure healthy lives and promote wellbeing for all at all ages:

- Target 3.3: By 2030, end the epidemics of AIDS, tuberculosis, malaria and neglected tropical diseases, and combat hepatitis, water-borne diseases and other communicable diseases.
- Target 3.d: Strengthen the capacity of all countries, in particular developing countries, for early warning, risk reduction and management of national and global health risks.

SDG 11 – Make cities and human settlements inclusive, safe, resilient and sustainable:

- Target 11.b: By 2020, substantially increase the number of cities and human settlements adopting and implementing integrated policies and plans towards inclusion, resource efficiency, the mitigation of and adaptation to climate change, resilience to disasters and develop and implement, in line with the Sendai Framework for Disaster Risk Reduction 2015–2030, holistic disaster risk management at all levels

Goal 13 – Take urgent action to combat climate change and its impacts:

- Target 13.1: Strengthen resilience and adaptive capacity to climate-related hazards and natural disasters in all countries.
- Target 13.3: Improve education, awareness-raising and human and institutional capacity regarding climate change mitigation, adaptation, impact reduction and early warning.
- Target 13.b: Promote mechanisms for raising capacity for effective planning and management in relation to climate change in the least developed countries and small island developing states, focusing on women, youth and local and marginalised communities.

*Source*: United Nations, 2015, *Transforming our world: The 2030 agenda for sustainable development,* United Nations Secretariat, New York

SDG, sustainable development goal.

Vector-borne diseases are threatening millions of people across the world and killing close to 700 000 individuals annually (WHO 2020). Diseases such as schistosomiasis, malaria, dengue fever, Chagas, African trypanosomiasis and lymphatic filariasis continue to weigh heavily on the global economy, causing about 17% of the global burden of diseases (Campbell-Lendrum et al. 2015). They cause debilitations and epidemics that disrupt health systems and cause a plethora of socioeconomic problems globally (Lozano, Naghavi & Foreman 2012; WHO 2008). Low-income countries and societies with low socioeconomic status are more vulnerable. In southern Africa, disease outbreaks are highly prevalent at endemic levels. The proliferation of VBDs and concerns about global climate change have given rise to questions about their potential relationship (Medlock & Leach 2015; Servadio et al. 2018; WHO 2014a). The diseases are sensitive to climatic conditions in different ways. Insect vectors that transmit diseases, and the pathogens and parasites that cause these diseases show sensitivity to temperature and precipitation (Servadio et al. 2018). Increases in temperature and highly variable weather patterns threaten to undermine recent global progress in the fight against VBDs (Campbell-Lendrum et al. 2015). These factors also affect the attainment of SDGs as they get in the way of economic growth and have a negative effect on the health of the masses and their general wellbeing (Braks et al. 2019; WHO 2017).

Servadio et al. (2018) state that mosquito-borne disease outbreaks occur with the highest frequency compared to other VBDs. Common diseases transmitted by mosquitoes include Chikungunya, malaria and dengue (Medlock & Leach 2015; Parham, Waldock & Christophides 2015; WHO 2014), which are getting widespread attention from researchers and medical practitioners. One of these diseases, malaria, is of great concern as it is common in economically depressed regions and contributes significantly to the failure of these regions to meet SDGs. In 2019 alone, about 229 million cases and 409 000 malaria-related deaths were reported (WHO 2021).

Mosquitoes are malaria vectors. They are known to breed under warm and wet conditions, and there is growing interest in exploring their sensitivity to temperature and precipitation patterns (Servadio et al. 2018). Scholarly work shows that higher temperatures are related to malaria outbreaks, but the composite dynamics between the environment, vectors and disease transmission require further scientific interrogation (Caminade, McIntyre & Jones 2019; Giesen et al. 2020; Gunda et al. 2017; Rohr & Cohen 2020). Studies by Mordecai et al. (2020), Shapiro, Whitehead and Thomas (2017) and Parham et al. (2015) show that extreme temperatures compromise the ability of vectors to transmit disease pathogens. This implies that the predicted rise of temperatures beyond 1.5 C by the end of the 21st century will likely reduce malaria incidences. However, other studies (Adekiya et al. 2020; El-Sayed & Kamel 2020; Moore 2021; Rohr & Cohen 2020) show that extreme temperatures will modify other environmental variables and create conditions that are conducive to the transmission of pathogens. Thus a rise in temperatures is likely to exacerbate the proliferation of malaria cases in the future. The divergent and complex views on the relationship between malaria and climate change reflect that there is still a not enough scientific understanding of how the disruption of biophysical and ecological systems might affect the longer-term wellbeing and health of populations. Consequently, the effectiveness of policies and actions to bring about the implementation of SDGs remains largely disputable. Therefore, any study that interrogates the relationship between climate change and malaria and the implications of this relationship for SDGs merit attention.

Malaria is expected to continue disrupting the attainment of SDGs as it is the most lethal VBD on the planet, affecting more than 109 countries on three continents (Africa, Latin America and Asia) (WHO 2021). In developed countries, several studies have been conducted on VBDs and climate change. These studies have largely been driven by the huge burdens that VBDs place on these countries, their high prevalence and their sensitivity to climate-related parameters (Campbell-Lendrum et al. 2015). In developing countries, VBDs are even more widespread, but scientific investigations in these countries are yet to proffer solutions. Despite the widespread occurrence of VBDs in Zimbabwe, little has been done in that country to determine whether they are the result of climatic vagaries or to investigate how their prevalence deters or promotes the attainment of SDGs. Localised studies are also scarce and those that are available focus mostly on rural areas. In Masvingo city, the impact of climate change on malaria and the implications of this interface for sustainable development remain unknown. Owing to this paucity of scientific knowledge, it is very challenging to implement local health and development strategies effectively. The current study sought to contribute to filling the knowledge gaps.

## Materials and methods

The study was conducted in Masvingo city, which lies in south-eastern Zimbabwe at 20.0720 S 30.8343°E. The city is close to the Great Zimbabwe Monuments, the national heritage site from which the country takes its name. The city comprises eight main suburbs from which data were collected, namely Mucheke, Victoria Range, Rujeko, Rhodene, Target Kopje, Zimre Park, Clipsham and Eastvale. The suburbs can be categorised as high-, middle- and low-density suburbs.

**FIGURE 1:**
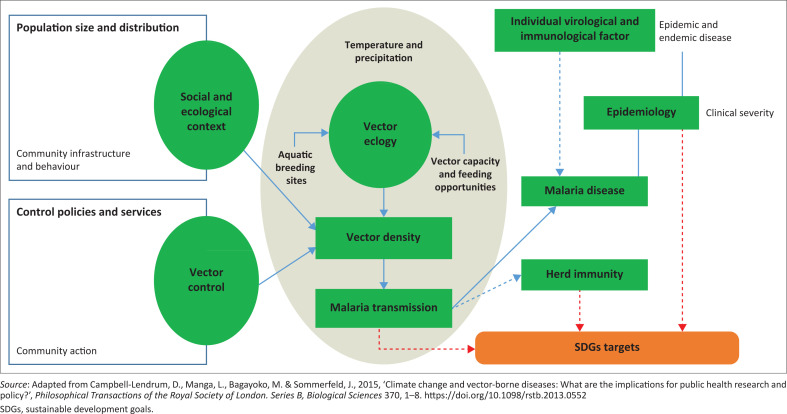
The climate–malaria interface and its link to sustainable development goals.

The study was premised on a concept that places meteorological variables at the core of VBD transmission (see Figure 1). It shows that climatic conditions (as determined by temperature and rainfall characteristics) are the key indirect determinants of the proliferation of VBDs (in this case malaria). Figure 1 shows that malaria transmission has the direct influence on SDG targets; its epidemism, endemism and clinical severity also have implications for the attainment of SDG targets.

Malaria, as well as some other VBDs, has aquatic developmental stages, which means that precipitation and related environmental conditions exert an important influence on pathogen transmission and consequently on disease prevalence. In the context of the conceptual framework, this study investigated how the changes in meteorological factors (temperature and precipitation) influence the proliferation of malaria and the implications of the proliferation of malaria for SDG targets.

### Research design and sampling

**FIGURE 2:**
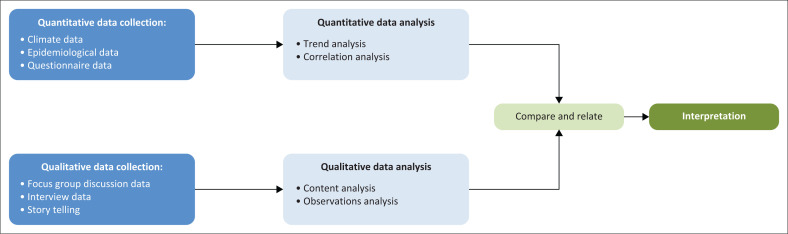
Mixed-methods approach.

A mixed-methods design that combined both quantitative and qualitative methods (König & Dreßler 2021) was used for this study. This approach connects and embeds the data and is generally regarded as a more comprehensive way of integrating all aspects of a programme of inquiry (Tashakkori & Creswell 2007). It mitigates the weaknesses of purely quantitative and qualitative methods by capitalising on the strengths of both (Chapungu 2017). For example, the quantitative approach may find a positive relationship between two variables (e.g. climate and VBDs), but this might not be accurate as correlation does not always mean causality. This gap can be filled by a qualitative approach that digs into the social dynamics contributing to existing patterns. Figure 2 summarises the methodological approach used in this study.

A mix of clustered random, purposive and chain-referral sampling techniques was used as strategies for identifying respondents in this study. The residential suburbs were treated as clusters from which households were randomly selected to participate in the study. The sample-size calculator of the Australian National Statistical Services was used for simple random sampling in clusters at a 95% confidence level. A total of 218 respondents were selected to participate in the study. The response rate was 87.5%. A final sample of 191 respondents participated in the survey. Purposive sampling was used in conjunction with the chain-referral approach to identify medical practitioners specialising in infectious diseases. Purposive identification of an expert in infectious diseases would lead to the identification of the next expert through a referral system, where an expert would refer the research team to another expert. This was done until a saturation point was reached, and the research team was referred to the experts already interviewed.

### Data collection

Questionnaire surveys were used for data collection. The questionnaires were administered in the residential suburbs at the household level. The surveys were complemented by key informant interviews conducted amongst purposively selected respondents and those identified through chain-referral sampling. The Climate Change Office and the Meteorological Services Department (MSD) were regarded as important stakeholders in climate change. Representatives of the Ministry of Health and Child Care of Zimbabwe provided key insights into the proliferation of VBDs in the city. An interview guide, which was basically a set of preconceived questions, was used to guide the interview process. The semi-structured interviews were used to obtain key informants’ views, which were based on their knowledge, expertise and experience regarding the impacts of climate change on VBDs. Some statistical data were obtained from the District Health Information System (DHIS) and the records of private and public health facilities.

Rainfall and temperature data were obtained from the Zimbabwe Meteorological Services Department (ZMSD). The datasets were complemented by records obtained from the National Climate Data Centre (NCDC) (http://www.ncdc.noaa.gov). A validation exercise was carried out to ensure that the data sets were compatible. This was performed through regression of the data sets. A Spearman rank correlation coefficient analysis revealed a strong positive relationship (*r* = 0.95) between the two data sets, with a coefficient of determination of 0.91. Given the strong positive relationship between the two data sets, the data from the two sources were combined and used for the analysis of climate change in the study.

Some statistical epidemiological data on the temporal and spatial patterns of VBDs were obtained from the Masvingo Provincial Hospital, seven local public clinics and five private health facilities in Masvingo city. From these, historical data on VBD cases were obtained.

### Data analysis

The data were analysed using exploratory and confirmatory data analysis approaches. Rainfall, temperature and malaria statistics, which constituted the time series data, were tested using the Kolmogorov–Smirnov test to ascertain whether they deviated from a normal distribution or not. Following this, the non-parametric Mann–Kendal (*M–K*) test was used to test the trends in precipitation, temperature and malaria prevalence statistics over time. The *M–K* test is a non-parametric method ordinarily used for detecting monotonic trends in series of hydro meteorological data (Pohlert 2016; Tehrani et al. 2021b). The test is simple and robust, and it can cope with missing values, seasonality and values below the detection limit (Kocsis, Kovács-Székely & Anda 2020). An add-in of Microsoft Excel, XLSTAT 2020, was used to carry out this test because of its ability to take into account and remove the effect of autocorrelations. If there are *n* observations *x*1; *x*2; … ; *xn* in the Mann–Kendall test, *S* is calculated from Equation 1:

$$S=\sum_{k=1}^{n-1}\sum_{j=k+1}^{n}sgn(Xj-Xk)$$ [Eqn 1]

where S is the Kendall score Sgn (*x*) = {1 if *x* > 0; 0 if *x* = 0; –1 if *x* < 0} (Mann 1945).

The variance of S is calculated from Equation 2:

$$Var(S)=\frac{1}{18}\left[n(n-1)(2n+5)-\sum_{p-1}^{g}tp(tp-1)(2tp+5)\right]$$ [Eqn 2]

where *ɡ* is the number of tied groups and *tp* is the number of observations in the *p*th group. After computing the variance, the *Z* value (*Z*_*mk*_) is computed in Equation 3:

$$\begin{array}{l} Z_{mk}=\frac{s-1}{\sqrt{VAR(S)}}\text{if }S\;>\;0\\ \;=0\text{ if }S\;=\;0\\ \;=\frac{s+1}{\sqrt{VAR(S)}}\text{if }S\;<\;0 \end{array}$$ [Eqn 3]

When the MK statistic *Z*_*MK*_ is positive, there is a positive change in the variable trend. Negative *Z*_*MK*_ indicates a decreasing trend in the studied variable (Tehrani et al. 2021a). The values greater than 1.96 and smaller than −1.96 indicate significant changes at a 95% confidence level.

#### Determination of period prevalence rate

The period prevalence rate for malaria was determined using Equation 4:

$$Prevalence=\frac{Number\;of\;Cases}{Total\;Population}\times 100$$ [Eqn 4]

### Ethical considerations

The permission to carry out the study was sought from the research ethics clearance committee at the University of South Africa, College of Agriculture and Environmental Sciences and Masvingo Provincial development committee in which the Ministry of Health and Child Care (MoHCC) is represented. The Helsinki Declaration Principles on human ethics were followed. The participating health facilities consented to the research processes. The participants in the study consented to participate voluntarily and were given the option to withdraw from the study at any stage. There was no coercion and no payments were made. The data collection tools were translated into local languages to enable effective participation by respondents.

## Results

### Climate change trends in Masvingo city: Focus on temperature and rainfall

Temperature and rainfall data for the past 40 years show that the climate of Masvingo city is changing. Table 1 contains data and descriptions of trends in eight bioclimatic variables identified.

**TABLE 1:** Bioclimatic variables explaining climate change in Masvingo city, Zimbabwe.

Climate change variable	*m + c*	*p*	Description of trend
MMMT	0.0327–38.399	0.001	Significant change/increasing trend
MMT	0.0187–17.651	0.002	Significant change/increasing trend
MTWM	0.863–89.854	0.011	Significant change/increasing trend
MinTCM	−0.0445 + 89.269	0.043	Significant change/declining trend
TAP	−4.7883 + 10116	0.049	Significant change/declining trend
MMP	0.4203 + 88534	0.046	Significant change/declining trend
PWQ	3.4206 + 7137.3	0.048	Significant change/declining trend
SMP	4.4614 + 60021	0.323†	Not significant/declining trend

MMMT, mean monthly maximum temperature; MMT, monthly maximum temperature; MTWM, maximum temperature of the warmest month; MinTCM, minimum temperature of the coldest month; TAP, total annual precipitation; MMP, mean monthly precipitation; PWQ, precipitation in the warmest quarter; SMP, seasonal mean precipitation.

† , Trend not significant at α = 0.05; *m + c* is the regression equation.

Seven of the eight assessed bioclimatic variables show a significant (*p* < 0.05) trend. As shown in Table 1, temperature variables indicate a warming climate at Masvingo city. The maximum temperature of the warmest month (MTWM), mean monthly maximum temperatures (MMMTs), minimum temperature of the coldest month (MinTCM) and mean monthly temperatures (MMTs) are all changing significantly. The declining MinTCM is indicative of weather extremities that come with climate change and variability. Precipitation variables also indicate a gradually drying city. The mean monthly precipitation (MMP), total annual precipitation (TAP) and precipitation in the warmest quarter (PWQ) show significantly declining trends. However, seasonal mean precipitation (SMP) shows no statistically significant trend although it is also declining like the other precipitation-related variables.

**FIGURE 3:**
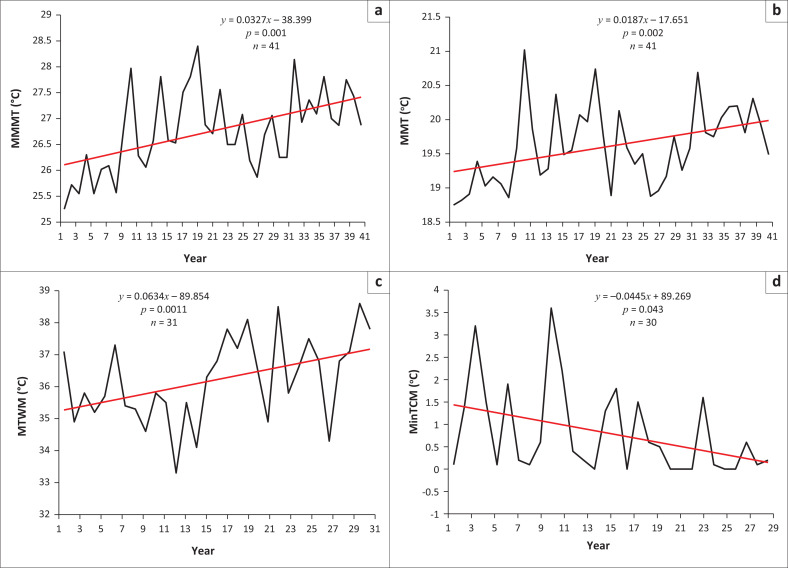
Temperature trends in Masvingo city, Zimbabwe: (a) mean monthly maximum temperatures (MMMT), (b) monthly maximum temperature (MMT), (c) maximum temperature of the warmest month (MTWM), and (d) minimum temperature of the coldest month (MinTCM).

Figure 3 shows long-term trends in temperature-related variables (MMMT, MTWM, MinTCM and MMT). The variables are key as they are indicative of major climatic shifts over time. In light of the influence of temperature on biological processes, the temperature-related variables are also important for determining the effects of climate change on VBDs.

**FIGURE 4:**
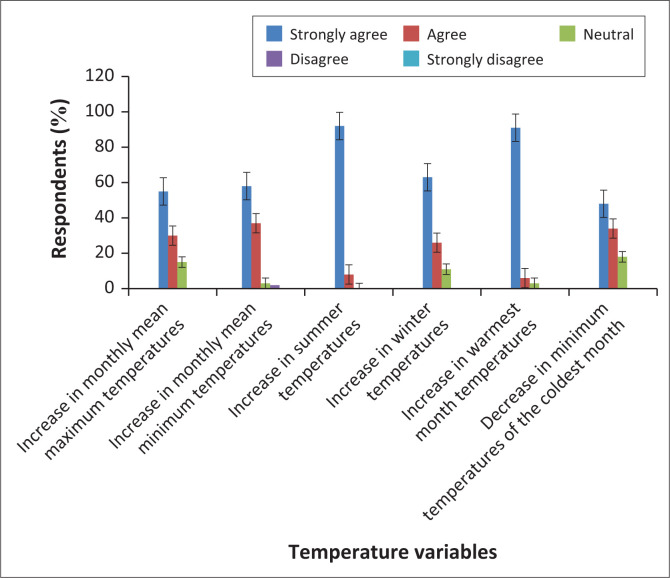
Perceptions of temperature changes in Masvingo city, Zimbabwe (*n* = 191).

As shown in Figure 3, the MMMT is changing significantly (*p* = 0.001, α = 0.05). The linear model presents an increasing trend. An increase of about 0.33 °C is estimated over the 40-year period. Over the period under investigation, MMT increased significantly (*p* = 0.002; α = 0.05). There was an approximate 0.27 °C increase in MMT over the study period. Figure 4 also indicates that MTWM statistically (*p* = 0.011; α = 0.05) increased over time. Based on a preliminary analysis of monthly temperature characteristics over 30 years, October is regarded as the warmest month of the year in this climatic region (Gwitira et al. 2014). The maximum temperatures for October are increasing, indicating a warming climate. In addition, MTCM shows that there was a significant (*p* = 0.043; α = 0.05) change in temperature regimes in the city. A qualitative analysis of temperature changes confirmed the validity of quantitative claims that there was a significant change in temperatures in the city. Figure 4 presents the views of local people and the percentage of respondents holding each view.

More than 50% of the respondents strongly agreed and more than 20% agreed that all temperature-related variables had been increasing over time. The remaining respondents were neutral. None of the respondents disagreed or strongly disagreed that temperature-related variables had changed over the period under study. Thus we observed that local people felt that temperature-related variables in the city had been changing over time. The people’s views confirmed observations based on an analysis of climate data recorded over time.

**FIGURE 5:**
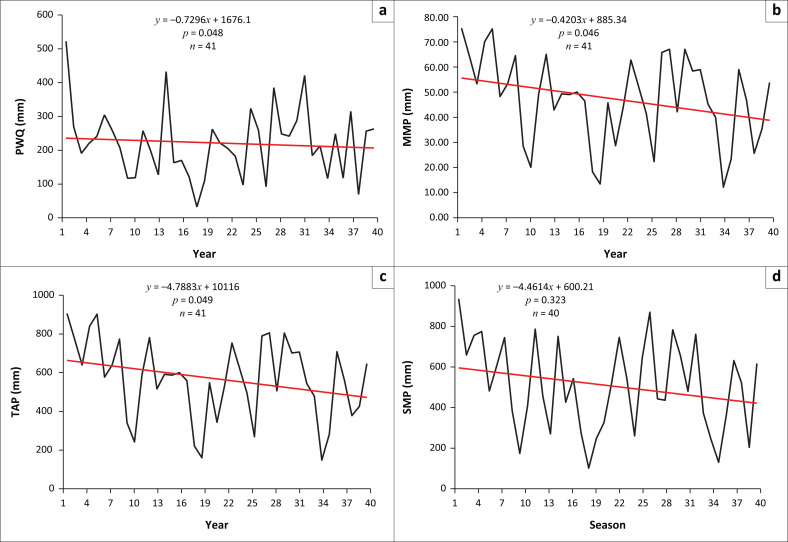
Precipitation in Masvingo city between 1974 and 2014: (a) Precipitation in the warmest quarter (PWQ), (b) mean monthly precipitation (MMP), (c) total annual precipitation (TAP) and (d) seasonal mean precipitation (SMP).

Precipitation variables such as the MMP, TAP, PWQ and SP were considered in the analysis of climate change in the city. Figure 5 shows that most of the variables were changing significantly, which indicates that the city had gradually been getting less precipitation over time. Besides the decrease, the trends exhibited high variability over time.

As indicated in Figure 5, the TAP trend is statistically significant (*p* = 0.049; α = 0.05). The MMP also shows a statistically significant (*p* = 0.046; α = 0.05) trend, which is reflective of a changing climate. The PWQ, which runs between October and December, also showed a significant (*p* = 0.048; α = 0.05) decline between 1974 and 2014. Whilst all other precipitation variables indicated statistically significant changes over time, the seasonal mean precipitation did not show a significant trend (*p* = 0.323; α = 0.05). However, it was observed that although not statistically significant, the trend had also been in decline over time.

The views of the local people confirmed the statistical observations. In general, respondents described the precipitation patterns in Masvingo city as unpredictable, highly variable and declining. About 73% (*n* = 191) of the respondents indicated that they strongly agreed that annual rainfall totals had decreased over time. 20% indicated that they agreed to this view, whilst 7% were not sure. The respondents reported an increase in the severity and frequency of droughts. An increase in the intensity of floods in the city was reported by 98% of the respondents. In general, variable descriptions by local people show that climate change is occurring, as indicated by the long-term changes in specific precipitation-related variables.

### The trend of vector-borne diseases in Masvingo city

**FIGURE 6:**
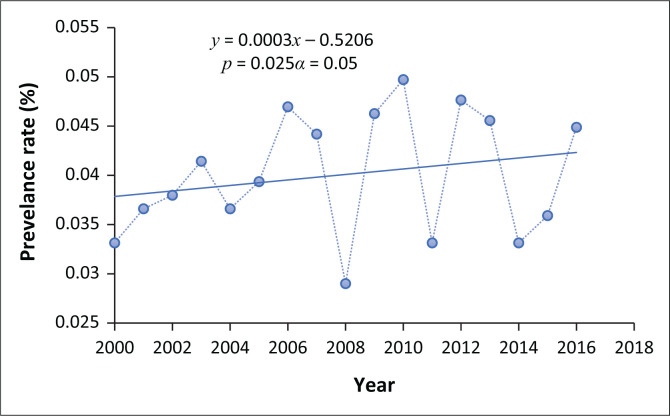
Malaria trends in Masvingo city.

Results show a significant (*p* = 0.025; α = 0.05) increase in the cases of VBDs recorded in Masvingo city (Figure 6). During the interviews, respondents reported that the city had not been prone to VBDs such as malaria before, but owing to changes in environmental conditions, the city was now vulnerable to these diseases.

Figure 6 shows that the cases of VBDs reported in Masvingo city fluctuated, but there was an overall increase in the long term despite the Ministry of Health and Child Care reporting in 2017 that significant progress had been made in reducing the incidence of malaria in the southern parts of Zimbabwe, where the city is located (MoHCC 2017). Periods of decrease could possibly be associated with the implementation of malaria control programmes in the city and nearby districts. The trend shown in Figure 6 does not reflect unreported cases. However, the data include all malaria cases recorded in health facilities and reported by respondents. It does not disaggregate according to imported, intraported, induced or autochthonous cases.

The emerging pattern is complex, and the fluctuations and overall increase cannot be explained by a single factor. Many confounding variables contribute to the disease dynamics. Results from the expert interviews indicate that a plethora of socioeconomic and environmental factors interact with and modify disease spatial and temporal patterns. One of the factors is inter-endemic area migration. It was reported that some of the cases seen at health centres had been ‘imported’ from areas such as Chiredzi and Mutare. However, local cases were reported to be existing and increasing despite the inefficiencies of the case detection and investigation systems. Other behaviours such as poor waste disposal promote the breeding of mosquitoes that may transmit malaria pathogens. An increased breeding rate elevates the chances of mosquito bites and consequently the probability of contracting malaria. Uncontrolled agricultural activities around the city were reportedly another factor that might have contributed to the increase in vector density. Swamps of mosquitoes were observed in crop fields and vegetated areas. Some respondents argued that there could be a relationship between increased population density and malaria cases, indicating that as population grows, it influences social, economic and ecological changes that may promote the occurrence of vectors that transmit diseases. However, it is a fact that temperature and precipitation regimes play a role in disease patterns given that insect vectors and parasites that transmit pathogens favour high temperatures and wet conditions.

### The relationship between temperatures and vector-borne diseases in Masvingo city

**FIGURE 7:**
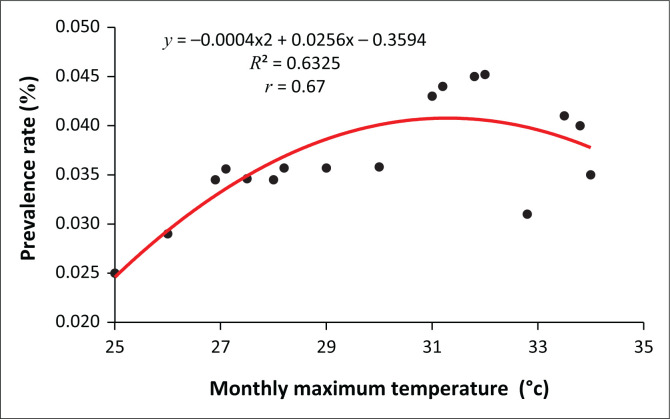
Relationship between the prevalence of malaria and mean monthly maximum temperature.

Although there are many factors that can explain the increased incidence of VBDs, our results indicate that mean maximum monthly temperature (MMMT) correlates positively with malaria cases and prevalence rates. As shown in Figure 7, there is a strong positive (*r* = 0.67; *r*^2^ = 0.63) relationship between the prevalence rate of malaria and MMMT.

As shown in Figure 7, the prevalence of malaria is highest at an MMMT of around 32 °C. However, as the MMMT continues to increase beyond 32 °C, the prevalence rate subsides. At a lower MMMT, the cases recorded were also low. Qualitative data show that there is a relationship between MMMT and the prevalence of malaria. More than 80% of respondents indicated that temperatures had been increasing along with an increase in malaria cases. One elderly respondent from Mucheke Suburb said the following:

We now experience more mosquito bites than before because temperatures have increased. To show that temperatures are responsible for more mosquito bites, during the cold season, there are no mosquitoes around here. If temperatures continue to increase, I see more cases of malaria-related fatalities in our neighbourhood in the near future.

The decrease in disease prevalence beyond 32 °C MMMT, as shown in Figure 7, does not mean that disease incidence will decrease if the temperatures continue to increase, because all the meteorological variables work in unison to create conditions that allow the proliferation of vectors responsible for transmission. The average weather conditions will determine vector population and pathogen transmission rates. High temperatures influence evaporation rate and consequently humidity, creating favourable hot and humid conditions.

### The relationship between precipitation and malaria prevalence

**FIGURE 8:**
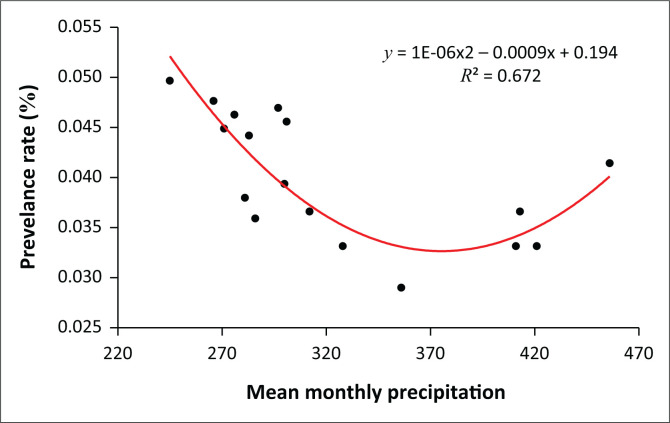
Relationship between malaria prevalence and mean monthly precipitation.

Results show that as the MMP decreases, the prevalence of malaria increases. Figure 8 shows this relationship. At an MMP of less than 270 mm, the prevalence rate is highest, but as precipitation increases, the prevalence rate decreases. However, above 320 mm, malaria cases appear to increase again.

A cross-correlation analysis (Table 2) of climate variables and prevalence rate shows that temperature and precipitation have a combined effect on the proliferation of falciparum malaria. Whilst there is a negative correlation between precipitation and prevalence rate, there is a positive relationship between temperature and the prevalence of malaria. Temperatures ranging between 30 °C and 32 °C provide the optimum conditions for malaria. In addition, an MMP ranging between 230 and 420 mm provides the optimum conditions for the occurrence of malaria.

**TABLE 2:** Relationships between temperature, mean monthly precipitation and malaria prevalence.

Variable	ToC	MMP	Prevalence
ToC	1	-	-
MMP	−0.39829	1	-
Prevalence	0.922724	−0.58432	1

MMP, mean monthly precipitation; ToC, temperature of the city.

This study shows that malaria prevalence in Masvingo city is partly influenced by climatic parameters. Consequently, the changes occurring in climatic parameters also influence changes in the prevalence of falciparum malaria. Thus, climate change has altered disease patterns over time. As temperatures increase, disease cases and prevalence also increase. This observation confirms the assertion of Campbell-Lendrum et al. (2015) that the changing climate, mainly the increase in temperatures, is related to an increase in the number of VBD cases. Thus VBDs are highly sensitive to climatic factors. Previous studies have shown that, unlike several health risks that are sensitive to climate change (e.g. exposure to storms, floods or heat stress), the influence of meteorological factors is less direct and more diverse both within and between individual diseases (Byrd et al. 2020; Wilcox et al. 2019). Temperature affects the biting, survival and reproductive rates of vectors, as well as the survival and development rates of the pathogens that they carry (Campbell-Lendrum et al. 2015).

Mean monthly precipitation has been shown to exert some influence on the proliferation of malaria, which is transmitted by vectors that have aquatic and humid developmental stages. Although this study shows that a decrease in precipitation is associated with an increase in the number of cases of malaria, previous studies (Campbell-Lendrum et al. 2015; Thomson et al. 2018) have shown that VBDs increase their abundance and spread when precipitation increases. This happens because vector insects and parasites favour high temperatures and wet conditions. Thomson et al. (2018) postulate that the negative effects of reduced precipitation and drought have been seen in Senegal, where *A. funestus* has virtually disappeared and malaria prevalence has dropped by more than 60% over the last 30 years. Our results could mean that other confounding variables besides MMP regulate pathogen transmission. A decrease in precipitation might result in a decrease in the amount of water in small water reservoirs, creating perfect conditions for mosquito breeding and the proliferation of parasites that transmit pathogens. Our study was also limited to the analysis of MMP and MMMT only. Other temperature and precipitation variables such as MMT, MTWM, MinTCM, PWQ, TAP and STP may also determine patterns of malaria prevalence.

It is the submission of this study that the changing climate exerts a range of more indirect yet extensive effects on the natural environment and on human systems. For instance, a decrease in precipitation amount may affect water storage, land use and irrigation practices. This may influence population movement, vector ecology and human exposure to infection. Some previously relatively stable geographical distributions are now changing because of a range of factors other than climate. These include water reservoirs, irrigation, population movements, rapid unplanned urbanisation, waste dumps and phenomenal increases in international travel and trade. A mix of environmental and socioeconomic factors may either underpin climate effects or neutralise them. Provided the complexity of these connections, it is not unexpected that there is plenty of observational evidence of the effects of meteorological factors on spatial, cyclical and inter-annual patterns of disease prevalence in specific locations.

## Discussion

This study shows that there is a change in the climate of Masvingo city, confirming earlier observations by Simba et al. (2012), Chapungu and Nhamo (2016) and Chapungu et al. (2020). The changes have influenced the temporal and spatial dynamics of vector-borne diseases.

Data from surveys on malaria prevalence in Zimbabwe are grouped at national and provincial levels. Therefore there are no detailed data on malaria prevalence at city level. Our study used city-level data obtained during the study period and various data collection methods. For climate variables, the study used MMMT and MMP. For malaria, the study computed the prevalence rate.

Our results indicate that there is a relationship between the changes in the selected climatic variables and the prevalence of malaria in the city. Research into the relationship between climate and malaria transmission has produced varied and conflicting results, showing the complexity of the relationship (Dhimal, O’Hara, Karki, Thakur, Kuch & Ahrens 2014; El-Sayed & Kamel 2020; Gunda et al. 2017; Mordecai et al. 2020). There appears to be no scientific consensus on this relationship so far. However, it is important to note that the studies focused on different geographical locations and used different climatic parameters, spatial scales and settlement types. Our results are in agreement with the observations made in the Gwanda District by Gunda et al. (2017) that malaria transmission rates are related to climatic variables. Several studies (Adekiya et al. 2020; Braks et al. 2019; Brand & Keeling 2017; Byrd et al. 2020; Fouque & Reeder 2019; MoHCC 2012; Rocklöv & Dubrow 2020; Rohr & Cohen 2020; Soko, Chimbari & Mukaratirwa 2015) have indicated that climate indeed has a significant influence on VBDs, including malaria.

We found that as temperature increases, the prevalence rate of malaria cases increases. The average prevalence rate for Masvingo city over the study period was 0.038%. The highest number of cases recorded per annum is 16 according to the records assessed in this study. This prevalence rate appears low, but what is frightening is that the number is increasing as the climate changes. It was also observed that as rainfall decreases, the prevalence rate of malaria increases. These findings are confirmed by a national scale study carried out by Egbendewe et al. (2011), which indicates that the average annual cases per 1000 in Zimbabwe increased to 98 between 1990 and 2000. A 1% increase in temperature was associated with an annual average increase of 12.56 in malaria cases. In contrast, a 1% change in precipitation resulted in a –0.53 per 1000 change in malaria cases. Epidemiological modelling shows that the optimal transmission of malaria occurs at average temperatures of 25 °C, and when mean temperatures rise beyond 28 °C, the rate of transmission decreases. However, this was not seen in this study. Our findings show that the highest prevalence rate is observed at 32 °C; beyond that, the prevalence rate starts to slow down. This is not surprising given the interplay and feedback mechanisms amongst socio-ecological factors that together influence malaria transmission rates. Mordecai et al. (2020) write the following:

The degree to which changes in climate suitability for disease transmission lead to changes in the incidence of disease depends on multiple factors, including pathogen exposure history, housing type, vector control and public health efforts, rainfall, and human mobility. (p. 420)

During the interviews conducted for this study, the provincial medical director indicated that some of the malaria cases recorded in Masvingo city were imported cases. Rural districts such as Chiredzi and Mwenezi are seeing more and more cases of malaria cases, and some of these cases are transferred to health facilities in Masvingo city. Patients who are mobile sometimes also travel to the city to be treated there instead of in their districts. This further confirms the impossibility of attributing malaria transmission and prevalence rates to meteorological factors alone. However, what is clear from our findings and those of previous studies is that changes that are taking place in meteorological variables (in our case MMMT and MMP) contribute to the modification of malaria transmission dynamics. The need to implement SDG 13 cannot be overemphasised, as it will go a long way to contain the proliferation of malaria in the city.

It is noteworthy that Masvingo city is not regarded as a malaria zone and most of the cases treated in the past at health facilities were imported cases. However, as the climate changes, conditions become more conducive to malaria transmission and more local cases are reported. This is in line with assertions by Ebi et al. (2005) who observed, using the MARA model, that climate change has the potential to alter the geographic distribution of malaria in Zimbabwe, with formerly unaffected areas with dense populations seeing more and more cases. The indications from the study that the number of cases is increasing should galvanise development practitioners in all sectors to take action to avoid possible disruptions associated with the proliferation of malaria in the city in the near future.

As the relationship between climate change and malaria affects the health of individuals, it has implications for the achievement of SDG 3, which includes the aim to end the proliferation of malaria. If the cases continue to increase as the climate changes, it means SDG 3 target 3.3 will not be achieved. Malaria-related illnesses have many spill-over effects that may indirectly determine the attainment of other SDGs and related targets. For example, lost productive time, resource use, debilitations and psychological effects may exacerbate poverty levels and compromise the attainment of SDG 1.

Attainment of SDG 11 could be affected by increasing cases of malaria as a result of the changing climate. Whilst the prevalence rate of malaria is considered to be low in Masvingo city, the increase in cases over the period under study is cause for concern. What it simply means is that the city now has areas that are conducive to the transmission of the disease and those areas are not safe. If any positive steps are to be taken towards achieving the sustainable city status, they should include actions to address the potential proliferation of VBDs in areas that are already prone to outbreaks of these diseases and take action to reduce environmental changes resulting from climate change.

The increase in malaria cases in Masvingo city signifies an increase in vulnerability to climate-induced hazards. Consequently, climate action, as specified by SDG 13, is of paramount importance. The results reveal that malaria could be driven by climate change. Therefore, there is need to strengthen resilience and adaptive capacity of the city and its people. As it stands, all signs indicate that resilience is low and the proliferation of malaria could further compromise the ability of the city to cope with climate-induced pressures.

## Conclusion and recommendations

This study shows that the climate of Masvingo city is changing – MMMTs are increasing and MMPs are decreasing. There is a relationship between the changing climate and the incidence and prevalence of malaria. Although correlation does not always mean causality, the relationships established in this study call for further interrogation of the matter and the implementation of precautionary measures, as the results show that climate change has a bearing on disease dynamics in the city. Even though the changing physical conditions and other socioeconomic variables that might contribute to malaria transmission and prevalence in Masvingo city remain unknown, we have shown that climate change is already playing a contributory role. However, we acknowledge the complexity of the matter given the divergent findings from previous studies in Zimbabwe and across the world. Importantly, we underline that the achievement of SDG targets for health, poverty, sustainable cities and climate action could be compromised by the interface between climate change and malaria. The need for action to address climate change through mitigation and adaptation measures cannot be overemphasised. The call for investment in the public health sector to ensure that it is climate proof is loud and clear. COVID-19 has demonstrated the need for a pragmatic public health delivery system, a clear understanding of health ecosystems and readily deployable emergency responses to improve the dexterity of communities to mitigate unanticipated infectious diseases. A climate-induced increase in VBDs is anticipated, and this calls for immediate efforts to promote climate change resilience to address the dynamics associated with infectious disease outbreaks.
